# Extended reality and emerging artificial intelligence in orthopedic surgical training: a scoping review of educational outcomes

**DOI:** 10.1007/s11701-026-03230-x

**Published:** 2026-05-25

**Authors:** Valentina Merino Molina, Rafael Andrés Barrera Medina, Johana Stefania Martínez Mora, Erwin Hernando Hernández Rincón

**Affiliations:** 1 Primary care physician, Universidad de La Sabana, Chía, Colombia; 2 Department of Family Medicine and Public Health, Universidad de La Sabana, Chía, Colombia; 3 Puente del Común, km 7 Autopista Norte, Chía, Colombia

**Keywords:** Virtual reality, Augmented reality, Mixed reality, Orthopedics and traumatology, Learning, Surgical training

## Abstract

Immersive technologies have gained increasing relevance in orthopedic surgical education; however, the scope, outcomes, and maturity of extended reality (XR) and artificial intelligence (AI) applications remain heterogeneous. To map the educational applications, learner populations, outcome measures, and research gaps related to the use of XR and emerging AI tools in orthopedic surgical training. A scoping review was conducted following Joanna Briggs Institute methodology and reported according to PRISMA-ScR guidelines. PubMed, Scopus, and ScienceDirect were searched for English and Spanish studies published between 2015 and 2025. Fifty-four studies involving 3,066 participants were included. Virtual reality (VR) was the predominant modality (83.3%), followed by augmented reality (31.4%), while AI-based applications were infrequently reported. XR-based training was most commonly evaluated in trauma surgery, arthroscopy, and arthroplasty, primarily among medical students and residents. Most studies reported improvements in simulation-based technical performance metrics, whereas evidence on clinical outcomes, long-term skill retention, and cost-effectiveness was limited. XR—particularly VR—represents the most mature immersive technology in orthopedic surgical education. AI applications remain emergent and primarily supportive. Future research should prioritize standardized outcome measures, multicenter designs, and evaluation of long-term educational and clinical impact.

## Introduction

The use of technologies in the field of medicine has experienced remarkable growth in recent years, with the implementation of virtual reality (VR) and augmented reality (AR) in various orthopedic surgery procedures, along with mixed reality (MR). In the field of biomedical engineering, these technologies are grouped under the term extended reality (XR) (Table 1) [1]. VR is a technology that uses three-dimensional images and databases to generate a simulated interactive environment, usually through a head-mounted display. AR, on the other hand, allows three-dimensional images reconstructed in real time to be superimposed on the patient’s anatomy via transparent hardware screens [2, 3]. 

**Table 1:** Definitions related to digital or virtual realities, which form part of the field of immersive technologies

Immersive technology	Definitions
Virtual Reality (VR)	General term referring to an artificially created, realistic, and interactive world [3]
Immersive Virtual Reality (IVR)	Contemporary term for VR that incorporates an immersive digital environment [3]
Augmented Reality (AR)	It allows three-dimensional images reconstructed in real time to be superimposed on the patient’s anatomy using transparent hardware screens [3]
Mixed Reality (MR)	It is a technology that combines VR and AR, merging the real and virtual worlds and allowing for immersive interaction between virtual and physical objects [42, 43]
Extended Reality (XR)	It is a general term used to refer to VR, AR, and MR [2]
Metaverse	Sum of all virtual environments. In its original definition, it was conceived as a single global virtual space, similar to the internet or the web [3]
Artificial Intelligence (AI)	Computational systems designed to perform tasks that typically require human intelligence, such as data interpretation, predictive analytics, decision support, and information management, when applied to educational or clinical contexts [61]
Low-fidelity simulation	Simulation modalities with limited anatomical and environmental realism, typically focused on isolated psychomotor skills or task-specific training [61]
High-fidelity simulation	Simulation environments that closely replicate real clinical scenarios, often integrating immersive technologies, anatomical realism, procedural workflows, and interactive feedback [61]

Source: authors’ own elaboration

The benefits of training orthopedic surgeons via VR simulation are multifactorial [4]. Conventional educational approaches involve mainly cadaver dissection, observations in the operating room, and simulation models. In contrast, VR offers the ability to replicate detailed anatomical structures and surgical procedures, allowing trainees to interact with and manipulate virtual models in real time [5]. As a result, its implementation has expanded to shoulder and knee procedures, as well as subspecialties such as sports traumatology and arthroscopic surgery, and even to postoperative settings in rehabilitation programs [6]. 

On the other hand, immersive technologies have various devices for each platform that allow interaction with the virtual environment. Among them, head-mounted devices (HMDs) are the most prevalent digital tools. For their implementation in orthopedics, multiple randomized clinical trials have been developed to evaluate their usefulness and advantages in orthopedic surgical training, thereby improving the knowledge and practical skills of the population undergoing surgery [5, 7, 8].

Although multiple systematic and narrative reviews have evaluated individual immersive technologies or specific orthopedic procedures, the rapid diversification of platforms, learner populations, and educational outcome measures has resulted in a fragmented evidence base. A scoping review is therefore required to comprehensively map current applications of extended reality and emerging artificial intelligence tools in orthopedic surgical training, identify commonly assessed educational outcomes, and highlight existing research gaps [9]. At the same time, educational development is essential in creating curricula structured around XR-based initiatives [4].

The objective of this scoping review was to map the educational applications, learner populations, assessed outcomes, and research gaps in the use of extended reality (VR, AR, MR) and emerging artificial intelligence tools in orthopedic surgical training.

## Materials and methods

**Fig. 1:**
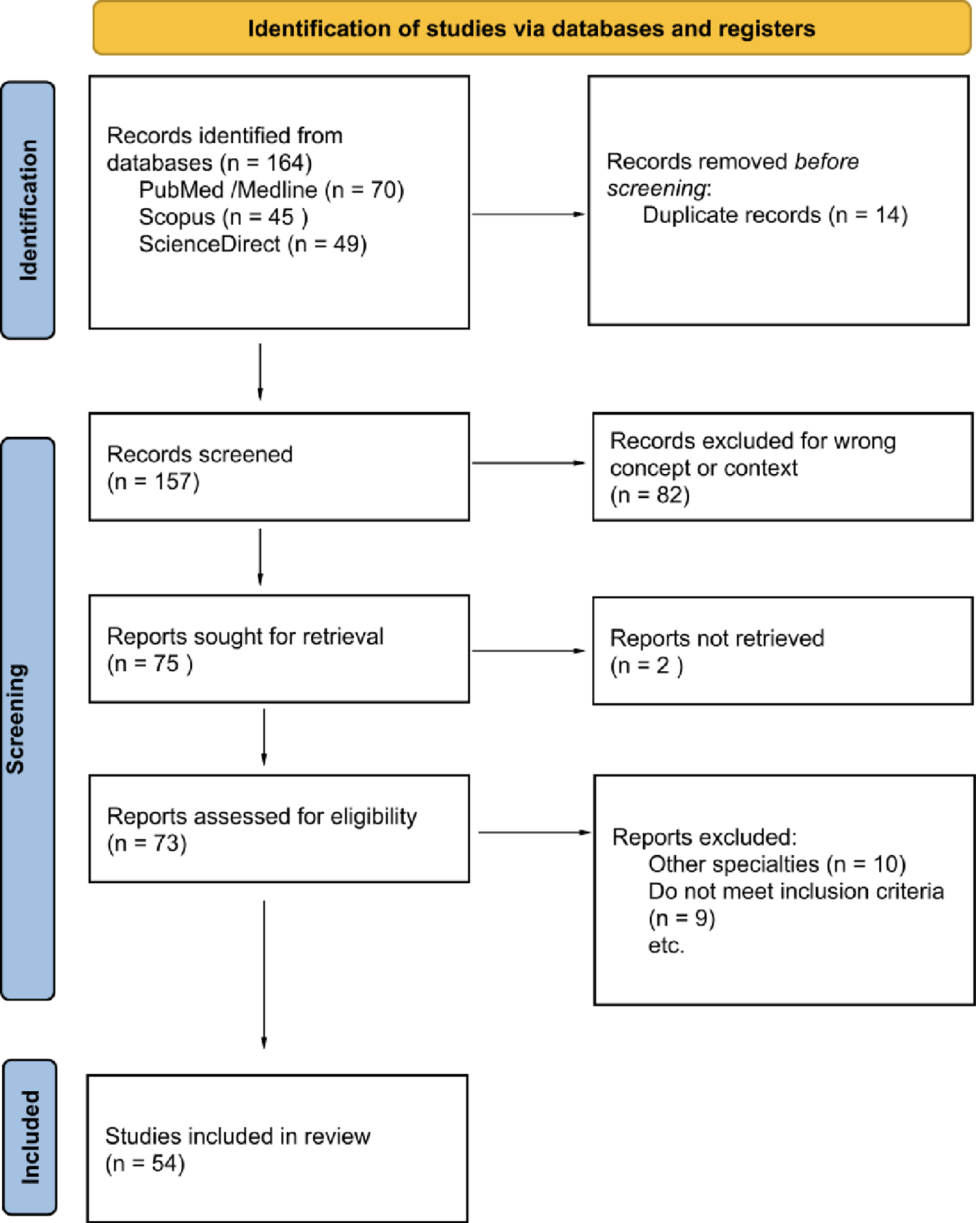
Methodology flowchart [PRISMA-ScR]. Source: authors’ own elaboration

A scoping review was conducted following the Joanna Briggs Institute (JBI) methodology and the PRISMA-ScR guidelines [Fig. 1] [10, 11]. Therefore, the central question was as follows: What is the evidence and how has augmented reality (AR) and artificial intelligence (AI) been applied in the surgical training of students, residents, and orthopedic surgeons, and what are its benefits, limitations, and areas of application?

The search strategy was subsequently defined. Relevant studies were identified and graphically represented. Finally, the results are summarized and presented.

In accordance with the Joanna Briggs Institute methodology for scoping reviews [10], review articles were not excluded a priori, as the objective was to map the breadth of educational applications and outcome domains. During data extraction, primary studies included within reviews were not double-counted in the quantitative synthesis to avoid duplication of evidence.

### Search strategy and data extraction

The search was limited to freely accessible full-text articles published between January 1, 2015, and September 1, 2025, and various sources of information were used. The databases included PubMed/Medline, Scopus, and ScienceDirect. This search was conducted with MeSH and DeCS terms such as “orthopedic training”, “orthopedic education”, “surgical education”, “augmented reality”, “virtual reality”, and “mixed reality”, using Boolean operators AND and OR were used to ensure adequate relevance to global literature data and findings.

### Study selection

The inclusion and exclusion criteria were defined to ensure the adequate selection of studies and their relevance [Table 2]. The references found in the databases described were transferred to Rayyan [12], a virtual platform designed for efficient research and information management in systematic reviews, where duplicates were subsequently removed manually. The studies were selected as follows:

**Table 2:** Search strategies for each of the selected databases and eligibility criteria

Eligibility criteria	
Inclusion criteria	Exclusion criteria
● Studies dealing with training in Orthopedics and Traumatology/Musculoskeletal Surgery (including arthroscopy, arthroplasty, trauma, orthopedic spine management, hand, foot-ankle, shoulder-elbow, etc.). ● The target population is: Medical students, orthopedic surgery residents/fellows, orthopedic surgeons in training/continuing education. ● Use of AR or VR tools and/or AI/LLMs (e.g., ChatGPT) for educational/assessment purposes in orthopedics (e.g., adaptive tutoring, automatic assessment, feedback, planning with educational outcomes). ● Publications between 2015 and 2025 ● Evaluation of educational/competency metrics using global scales. ● Studies must be in English/Spanish.	● Studies dealing with any nonorthopedic area (e.g., general surgery, urology, gynecology, nonmusculoskeletal neurosurgery) or rehabilitation of patients without an educational-surgical focus. ● Studies that deal with technical tools without an educational component ● Total absence of educational outcomes (only technical or hardware description without learning assessment) ● Total absence of educational outcomes (only technical or hardware description without learning assessment) ● Editorials/letters, opinions without data; conference abstracts without full text; purely technical studies without an educational component; series of patient cases without educational evaluation. ● No full text available when it prevents the extraction of educational methods/outcomes
**Search Strategies**	
**Data bases**	**Health Science Descriptors (DeCS) and Medical Subject Headings (MeSH) terms used**
PubMed	(“orthopedic training“[Title/Abstract] OR “orthopedic education” OR “surgical education”) AND (“Augmented Reality” OR “virtual reality” OR “mixed reality”)
Scopus	TITLE-ABS-KEY (“orthopedic training” OR “orthopaedic training” OR “orthopedic education” OR “orthopaedic education” OR “orthopedic surgical training” OR “orthopaedic surgical training” OR “orthopedic surgery education” OR “orthopaedic surgery education”) AND TITLE-ABS-KEY (“augmented reality” OR “virtual reality” OR “mixed reality” OR “artificial intelligence” OR “machine learning” OR “deep learning”) AND PUBYEAR > 2014 AND PUBYEAR < 2026 AND (LIMIT-TO (DOCTYPE, “re”) OR LIMIT-TO (DOCTYPE, “ar”)) AND (LIMIT-TO (LANGUAGE, “English”))
Sciencedirect	(“orthopedic training” OR “orthopaedic training” OR “orthopedic education” OR “orthopaedic education”) AND (“augmented reality” OR “virtual reality” OR “artificial intelligence” OR “machine learning”)

Source: authors’ own elaboration

Initially, three researchers independently examined the articles by title and abstract to determine their relevance, and any discrepancies were resolved among the groups of researchers. On the basis of an in-depth reading of the selected literature, data extraction was performed in a table previously structured by the research team. This table included the lead author and year of publication, country where the study was conducted, type of study, number of participants, procedure to be performed, type of participants, type of resource and device used, training area vs. comparator, and important results. Given the heterogeneity, we performed a narrative synthesis.

## Results

A total of 164 records were identified in the initial database search; 14 were duplicates. A total of 110 studies that did not meet the eligibility criteria were excluded during the screening phases. Consequently, 54 studies (total *n* = 3,066 participants), published between 2015 and 2025, were included. A publication trend was identified from 2018 onward, with the highest number of publications in 2024 (15 studies), followed by 2022 (8 studies) [Table 3].

**Table 3:** Articles included in the review

Lead author, year, country	Type of study	Evaluated technology	Assessed Task and Outcome Assessment
Bartlett J, et al. [42] 2021 United Kingdom	Pre–Post (questionnaires)	VR = Arthroscopic VR simulator (device NR; ArthroS/Simbionix mentioned)	Arthroscopy camera handling/diagnostic exploration (hip). VR exposure can stimulate interest in orthopaedic surgery and arthroscopic; feasible without direct supervision
Feeley A, et al. [44] 2022 Ireland	Crossover randomized trial	VR = PrecisionOS (HMD VR)	Cephalomedullary nail procedure workflow. Consider supervision effects on stress/performance when designing curricula Performance in simulation-based tasks correlated highly with a level of experience
Walbron P, et al. [33] 2020 France	Prospective comparative	VR = VirtaMed ArthroS – FAST modules (Periscoping, etc.)	Arthroscopy camara handling/economy motion. Investments made by a training center are more beneficial to arthroscopy beginners than occasional one-off training sessions, which allows them to have regular training and maintain the acquired skills over time
Berhold D et al. [19] 2022 USA	Systematic review	VR = Various HMD platforms (review)	THA, RSA, pedicle screw, glenoid exposure, tibial intramedular nailing, UKA. 6/8 RCTs favored VR-HMD over traditional training
Calem D, et al. (37) 2024 USA	Review	Mixed Reality (MR) = HoloLens and MR platforms (review)	Planning, navigation, remote proctoring. Potential improvements; heterogeneous evidence
Lorhe R, et al. [45] 2020 Canada	Narrative review	VR and AR	Arthroscopy, arthroplasty upper extremity. VR/AR adoption growing; need standardized validation
Sun P, et al. [38] 2023 China	Systematic review	VR and AR	Preop planning, intraop navigation, rehab. Promising across domains; more comparative data needed
Reinhold M, et al. [28] 2024 Germany	Randomized controlled trial	VR = Meta Quest 2 VR classroom + OR platform	3D lumbar anatomy; pedicle screw steps; supervised VR practice. DOPS higher; similar theory; better innovation perception
Cevallos N, et al. [46] 2022 USA	Randomized pilot	VR = Osso VR (Oculus) – SCFE module	Guidewire placement under simulated fluoro. Favorable trends in other metrics with VR training, less simulated damage
Lohre R, et al. [3] 2022 USA	Narrative review	VR/AR = Head Mounted Device with VR; HoloLens/Magic Leap	Anatomy, planning and surgery guidance. Summarizes benefits and curriculum frameworks
Zhou X, et al. [36] 2024 China	Review	VR + Robotics (planning/navigation) = MAKO/ROSA/Mazor/Navio/Velys/Versius	Preoperatory planning, intraoperatory navigation, reduction with robots, assisted cut by robots. Learning curves are satisfactory with significant procedures
Scott W, et al. [32] 2024 United Kingdom	Cohort study	Box Simulator = ArthroBox + USB scope	Arthroscopy triangulation. Augmented interest in training, better results oon those who play videogames
Redondo C, et al. [31] 2020 USA	Randomized controlled trial	Box Simulator = ArthroBox	Basic arthroscopy triangulation skills. ↑ ASSET knee + safety with simulator training
Woodward C, et al. [47] 2025 United Kingdom	Systematic review	VR/AR = ArthroS, ArthroBox, ARTHRO Mentor.	Multiple VR and AR simulators were compare. Acceptability and skill gains with simulators
Chung M, et al. [43] 2025 China	Comprehensive review	VR/AR + serious games = GBL platforms and VR modules	Inmersive simulation. Engagement and skills benefits; barriers remain through accesibility
Zhang B, et al. [15] 2022 USA	Prospective	Box Simulator = ASSH STEP psychomotor tool/modules	Screw fixation, depth of plunge, skin graft, wrist artrhoscopy. Training group improved and outperformed control on key metrics
Mok T, et al. [20] 2021 China	Randomized controlled trial	VR = VR tendon repair simulator	Tendon repair suturing technique = VR group scored higher on global ratings vs. control
Mensah E, et al. [39] 2024 USA	Narrative review	IA/AR/VR	Anatomy, planning, intraop guidance (overview). Highlights learning curves, tech hurdles; advocates simulation
Vergis A, et al. [48] 2018 Canada	Review	VR + box trainers = FLS/GOALS frameworks; simulators.	Skill acquisition & assessment. Simulation improves skills; FLS/GOALS validated
Azher S, et al. [49] 2024 Canada	Systematic + scoping review	VR (with/without haptics)	Multiple surgical tasks. Majority favored haptics; standardization needed
Walshaw J, et al. [50] 2025 United Kingdom	Systematic review	VR + robotic platforms	Multi‑specialty robotic skills. Core components: didactics, dry lab, VR; gaps in validation
Crockatt W, et al. [16] 2023 USA	Randomized controlled trial	VR (iVR) = Head‑mounted iVR vs. cadaver lab	rTSA steps/glenoid/baseplate workflow. iVR comparable/beneficial vs. cadaver
Abdullah A, et al. [51] 2024 Saudi Arabia	Systematic review	AR	Psychomotor tasks; anatomy; procedural steps. AR generally positive; heterogeneity noted
Zhang J, et al. [34] 2023 United Kingdom	Systematic review	VR + AR	Preoperative planning, intraop guidance/imaging. Patient satisfaction, postoperative complications, operating time, lenght of hospital stay
Pierzchajlo N, et al. [29] 2023 USA	Narrative review	AR = XVision, HoloLens, ImmersiveTouch	MISS planning and intraoperative guidance. Potential risks and complications from AR MISS
Forgione A, et al. [52] 2017 Italy	Systematic review	VR/AR = FLS/OSATS/box/wet-lab/VR	Skills acquisition & assessment frameworks. Supports simulation-based education; needs robust validation
Tung W, et al. [53] 2024 USAA	Systematic review	Gamification/Serious Games (VR/AR context)	Cadaveric carpal tunnel release, total knee arthoplasty, open reduction and internal fixation of ankle fracture, knee arthoscopy (residents). Engagement ↑; skills may improve; heterogeneity persists
Nasar A, et al. [54] 2021 USA	Systematic review & meta-analysis	VR simulators	Technical/psychomotor skills. VR generally improves technical performance vs. controls
Kayaalp M, et al. [6] 2025 Turkye	Narrative review	XR/Metaverse (AR/VR/MR)	Training, arthroscopy, arthroplasty, rehab. Describes XR use-cases and potential benefits/limits
Cate G, et al. [4] 2023 USA	Scoping review	VR = Simbionix ArthoMentor, TraumaVision	Curriculum, validation, skills training. Focus on simulator validation
Longo U, et al. [40] 2024 Italy	Systematic review	AR navigation; VR training modules	Component positioning; surgical planning; training Improved glenoid accuracy and screw length
Li T, et al. [5] 2025 China	Systematic review & meta-analysis	VR = Head-mounted VR and task VR simulators	Knowledge; operative skills; surgical design. Knowledge, clinical operation and surgical design scores, clinical understanding ability, clinical thinking, teaching interest and satisfaction
Coxe F, et al. [55] 2025 USA	Narrative review	VR = ArthroS, OssoVR, PrecisionOS	Immediate skill & knowledge acquisition. Procedure specific checklists, objetive grading scales, time to task completion, accuracy of implant placement
Mandal P, et al. [21] 2022 India	Narrative review	AR/VR	TKA planning/positioning; training. Potential gains in precision and training
Sugand K, et al. [22] 2018 United Kingdom	Multicenter study	VR = TraumaVision VR FlouroSim	Dynamic hip screw procedure (guidewire; placement) − 1 attempt. Experts/intermediates outperform novices
Wilson G, et al. [23] 2020 United Kingdom	Randomized crossover trial	VR = Google Cardboard VR vs. physical models	Fracture fixation principles; osteotomy concepts. Both improved; no significant difference VR vs. physical models
Bajuri M, et al. [56] 2021 Malaysia	Systematic review	VR	Curriculum & simulator design factors. Identifies key CSFs affecting effectiveness and adoption
McKnight R, et al. [57] 2020 USA	Narrative review	HMD VR; AR	Training frameworks; ethics; device landscape. Describes benefits and limitations; highlights standardization needs
Bartlett J, et al. [30] 2018 United Kingdom	Validation	VR = Hip arthroscopy VR simulator	Arthroscopy camera handling/diagnostic tasks. Sufficient face validity demonstrated
Wu L, et al. [24] 2024 Switzerland	Experimental analysis	AR/VR = AR HMD, HoloLens 2	Femoral neck osteotomy. UI and visualization of AR guidance can impact training experience
Hiemstra L, et al. [7] 2024 Canada	Cross-sectional observational study	VR = iVR system	Surgical training, procedure planning, and remote education/mentorship. Paper-based questionnaire with 13 Likert-scale questions; agreement percentages reported by domain (teaching, clinical, remote education)
Gupta N, et al. [41] 2024 India	Narrative review	AI	Orthopedic education and training. Descriptive evaluation of potential applications, benefits, limitations, and challenges associated with the use of Generative AI in orthopedics
Vaughan N, et al. [58] 2016 United Kingdom	Review	VR	Simulator taxonomy; validation evidence. Identifies gaps (e.g., THR training) and validation needs
Chiedozie K, et al. [25] 2023 Slovenia	Narrative review	VR	Bone trauma tasks. Narrative synthesis of VR applications in trauma
Unal Y, et al. [8] 2024 Turkey	Prospective	VR = Head‑mounted VR training (pre‑OR)	TKA procedural steps. VR group outperformed traditional on key OSATS domains
Blumstein G, et al. [17] 2020 USA	Randomized controlled trial	VR = Oculus Touch Motion controllers	the SawBones procedure. The VR group performed procedures significantly faster, completed more correct steps, and achieved higher global assessment scores
Longo U, et al. [35] 2021 Italy	Systematic review	AR/VR/IA	Education; planning; intraop assistance, surgical trainning. VR strong for training; AR improves precision; AI mostly diagnostic
Kuhn A, et al. [26] 2024 USA	Qualitative original research	VR	General surgical simulation concepts. VR is more useful for interns and junior residents. Benefits in anatomy, surgical exposures, and the steps of a procedure before seeing it for the first time
Lamb A, et al. [59] 2022 USA	Prospective, blinded study	VR = Osso VR	Learning phase and mock surgery. Statistically significant difference in favor of the VR group regarding time to completion and the number of redirections
Berezowsky C, et al. [27] 2022 Mexico	Implementation report (course)	VR = Quest 2 (immersive VR) at AO course	Nailing procedure steps (course setting). High realism (~ 62%), usefulness (76%), and intention to use (> 90%)
Hasan L, et al. [2] 2021 USA	Narrative review	VR	Arthroscopy; trauma; arthroplasty. Benefit to using virtual reality as a training tool, including in areas such as arthroscopy, arthroplasty, and trauma
Clarke E, et al. [9] 2021 United Kingdom	Systematic review	VR = ArthroSim, ArthroVR, insightArthro simulators	Execution time, surgical dexterity, technical accuracy. 15/16 studies determined that trainees using VR simulations perform better than those using standard training methods
Morya V, et al. [18] 2024 Korea	Narrative review	IA = ChatGPT	Virtual surgical simulation, intraoperative guidance, surgical planning. Highlights potential across perioperative continuum; warns on bias, hallucinations, ethics
Chatterjee S, et al. [60] 2023 Korea	Narrative review	IA = ChatGPT/LLMs	Guidelines in orthopedic surgery, Disease diagnosis in assisting surgeons, and research workflows. Evaluate how ChatGPT and other LLMs can enhance education, surgical planning, research, and data management in orthopedics

Source: authors’ own elaboration

Among the included records, narrative reviews predominated (12 studies, 22%), followed by systematic reviews (11 studies, 20%). Randomized studies were less frequent (5 studies, 9%). Most studies involved mixed cohorts, including orthopedic residents, medical students, and specialists [13–15].

**Fig. 2:**
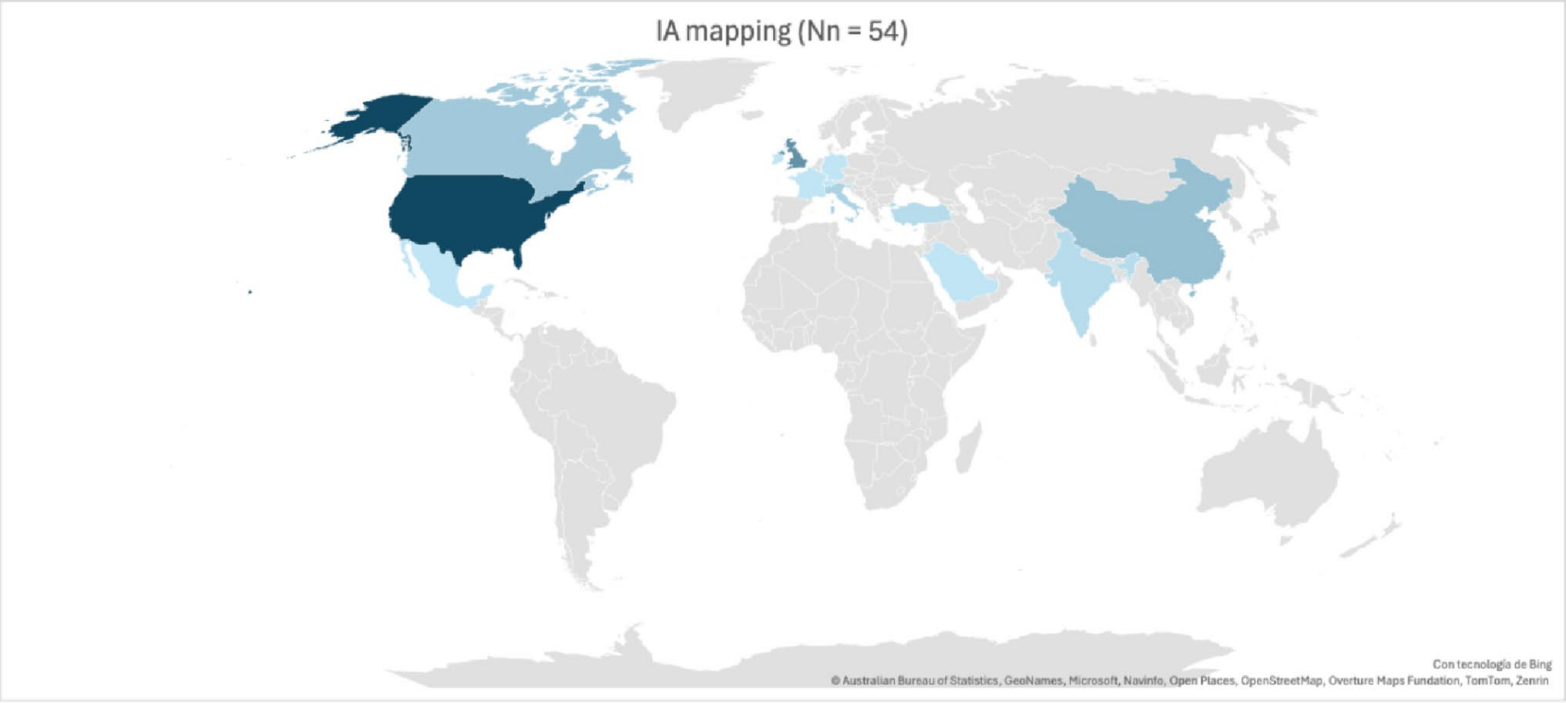
Geographical distribution of the included studies. The United States of America is the country with the highest number of studies [18] followed by the United Kingdom [10], China [5], Canada [4], Italy [3] Turkey [2], India [2] and Korea [2]. Some studies included multiple European countries [5]. Source: authors’ own elaboration

Publications originated worldwide, with the United States contributing the largest number (18 studies, 33%), followed by the United Kingdom (7 studies, 13%). Most studies were conducted in high-income countries across Europe, Asia, and North America [Fig. 2].

Across the included studies, 3,066 participants were analyzed. Orthopedic residents (1,238 participants; 5 studies) and medical students (817 participants; 9 studies) were the most frequently represented groups, followed by mixed cohorts (332 participants; 7 studies). Interns (46 participants; 2 studies) and surgeons or fellows (26 participants; 1 study) were less commonly included.In several studies, participant type was not explicitly specified. In several studies, the terms “interns” and “residents” were used interchangeably or without clear differentiation.

1. **Immersive technology and artificial intelligence tools**.

Virtual reality was the most frequently used modality in orthopedic training (*n* = 45, 83.3%), followed by augmented reality (*n* = 17, 31.4%), while artificial intelligence applications were less commonly reported (*n* = 5, 9.2%) [9, 16].

2. **Training tasks and results assessment**.

Training tasks were grouped into three main domains: orthopedic surgical procedures, arthroscopy, and surgical planning.

2.1. **Orthopedic surgical procedures**.

**Fig. 3:**
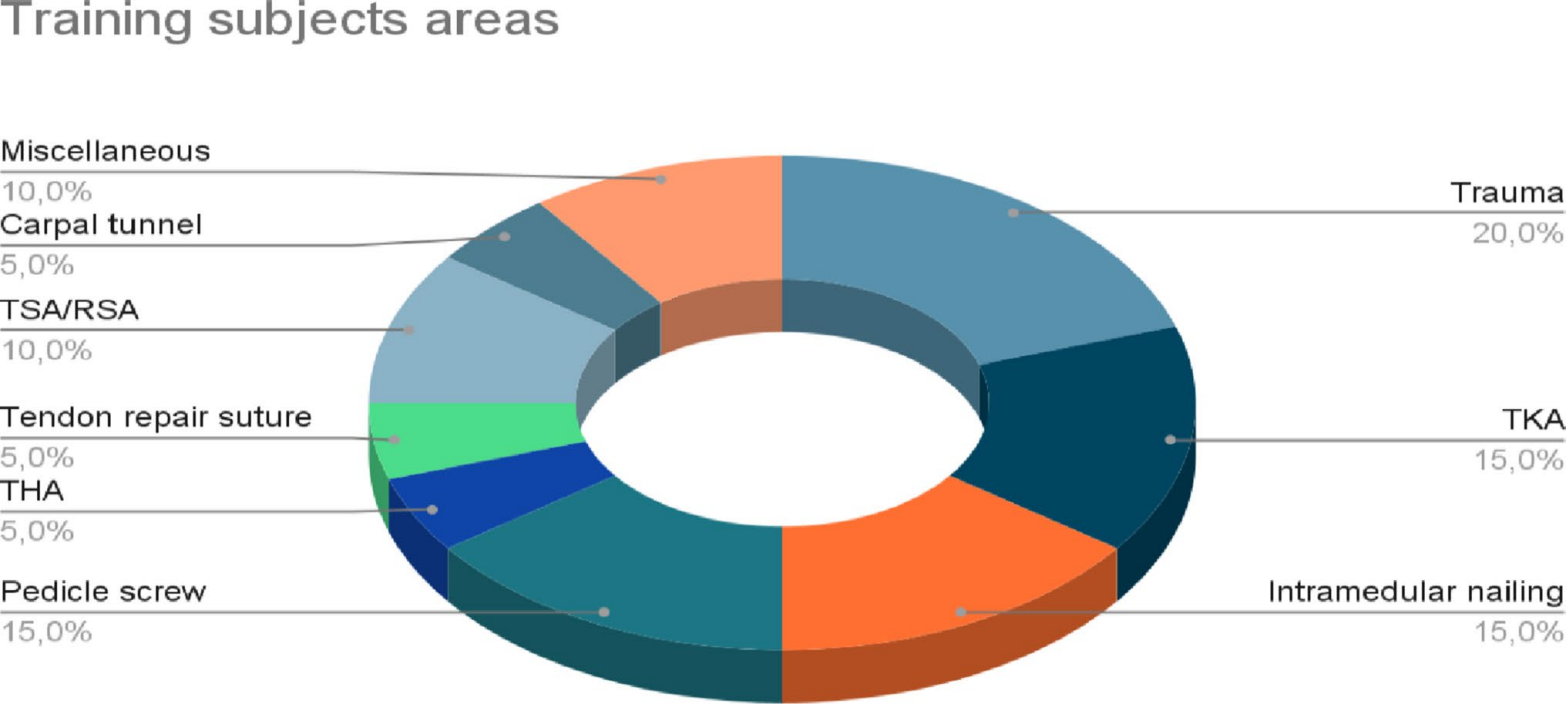
Training subjects areas. TKA = total knee arthroplasty, TSA/RSA = total and reverse shoulder arthroplasty, THA = total hip arthroplasty. Source: authors’ own elaboration

A wide range of interventions was reported, including trauma surgery, joint replacement procedures, and general surgical principles. The most commonly evaluated procedures included orthopedic trauma (*n* = 4), total knee arthroplasty (*n* = 3), intramedullary nail osteosynthesis (*n* = 3), and pedicle screw placement (*n* = 3). [Figure 3].

Across trauma- and arthroplasty-related procedures, randomized and multicenter studies reported that VR-based training was associated with improvements in simulation-based performance metrics, including procedural accuracy, task completion time, and global rating scales. These effects were more consistently observed among novice learners, while differences were less pronounced among intermediate and expert surgeons [17–20].

In trauma-related training, several randomized studies evaluated VR-based simulators for fracture fixation and intramedullary nailing, reporting higher procedural accuracy and improved technical performance compared with conventional training methods [23]. A prospective study involving 20 orthopedic residents with no prior experience in trauma-related arthroplasty or VR training reported significantly higher OSATS scores in the VR-trained group compared with controls [8].

2.2. **Arthroscopy**.

Arthroscopy training tasks were categorized into three main domains, with camera handling and diagnostic exploration considered a single integrated category.

Across randomized and prospective studies, VR and low-fidelity simulators were primarily used to develop fundamental arthroscopic skills, including hand–eye coordination, camera control, and motion economy. Trainees demonstrated improvements in execution time, instrument handling, and global assessment scores compared with baseline or control conditions [28–31].

3. **Preoperative planning**.

A subset of studies (*n* = 7) evaluated immersive tools for preoperative planning. These systems were primarily used to enhance anatomical understanding, procedural orientation, and workflow preparation [16]. Studies reported associations with improved spatial orientation, reduced radiation exposure, and fewer technical errors during simulated or early clinical procedures when VR or AR platforms such as Magic Leap, XVision, and ImmersiveTouch were used [33, 34].

Preoperative planning applications were reported across multiple procedures, including shoulder arthroscopy, hip arthroplasty, and spinal surgery. These tools enabled three-dimensional visualization of patient-specific anatomy and supported implant selection and portal planning [35, 36].

4. **Artificial intelligence applications**.

Artificial intelligence applications, as defined in this review, were infrequently reported and were primarily described in supportive roles, such as procedural planning, predictive analytics, and information management. Most AI-related studies were descriptive in nature, with limited empirical evaluation of educational outcomes [16, 37].

## Discussion

This scoping review mapped the current evidence on the use of extended reality (XR) and emerging artificial intelligence (AI) applications in orthopedic surgical training. The findings demonstrate a clear predominance of virtual reality–based educational interventions, with augmented reality used more selectively and artificial intelligence applications remaining limited and primarily supportive [9, 16, 37]. Overall, XR technologies have been most frequently evaluated in simulation-based environments, with outcomes focused on technical skill acquisition rather than clinical or patient-level endpoints [17–20, 23].

Across the included studies, virtual reality emerged as the most mature and widely adopted immersive modality in orthopedic education [2, 9, 17]. VR-based training was consistently associated with improvements in simulation-based performance metrics, particularly among novice learners, including procedural accuracy, task completion time, and global rating scales [8, 17–20, 23]. These findings align with educational models emphasizing deliberate practice, repetition, and immediate feedback during early stages of surgical skill acquisition [9, 39]. In contrast, the benefits of immersive training were less pronounced among intermediate and expert surgeons, suggesting a potential ceiling effect once foundational psychomotor skills are established [20].

It is important to note that comparator heterogeneity was substantial across the included studies. In some cases, virtual reality platforms were used as the reference standard rather than being compared with cadaveric training or physical simulators, which may introduce bias in favor of immersive technologies and limits direct comparison across educational modalities [31].

Augmented reality applications were more commonly reported in procedure-specific contexts, particularly in preoperative planning and intraoperative guidance [33–36]. AR platforms were primarily used to enhance spatial understanding, anatomical orientation, and workflow preparation rather than to deliver standalone training curricula [32, 35]. Although several studies reported associations with improved technical precision and reduced radiation exposure, the heterogeneity of study designs, procedures, and outcome measures limits direct comparison across technologies and clinical contexts [33, 34].

Preoperative planning represented a distinct and growing application domain for immersive technologies. XR-based planning tools were reported across multiple orthopedic subspecialties, including shoulder arthroscopy, hip arthroplasty, and spinal surgery, enabling three-dimensional visualization of patient-specific anatomy and procedural workflows [35, 36]. These applications were associated with improved spatial orientation and technical preparedness in simulated or early clinical settings; however, most evidence remains limited to short-term outcomes, underscoring the need for standardized evaluation frameworks and longitudinal assessment [16, 32].

Artificial intelligence applications were infrequently represented in the included literature and were primarily described in supportive roles, such as information management, predictive analytics, and procedural planning assistance [16, 37]. Unlike XR technologies, AI tools were rarely evaluated using structured educational outcome measures or validated assessment frameworks. This finding highlights the early developmental stage of AI integration in orthopedic education and emphasizes the need for empirical studies assessing its educational effectiveness, reliability, and ethical implications before widespread curricular adoption [37, 41].

From an educational perspective, the findings of this review suggest that XR technologies are best positioned as adjuncts to traditional orthopedic training rather than replacements [4, 9, 26]. Immersive simulation offers a controlled and reproducible environment that supports competency-based progression, particularly for novice learners [8, 17, 23]. However, variability in training duration, lack of standardized outcome measures, and limited reporting of long-term skill retention remain important barriers to consistent curricular integration [4, 39, 47].

Several research gaps were identified. First, most studies focused on short-term simulation-based performance outcomes, with limited evaluation of skill transfer to the operating room or long-term retention [17, 20, 55]. Second, formal economic evaluations assessing cost-effectiveness, maintenance costs, and scalability were infrequently reported [3, 55]. Third, the predominance of studies conducted in high-income countries limits generalizability to resource-constrained settings [27]. Finally, the educational role of artificial intelligence remains underexplored in comparison with more established XR modalities, representing an important direction for future research [37, 41].

Consistent with principles of simulation-based education, the effectiveness of immersive technologies is influenced not only by the technology itself but also by structured briefing and debriefing processes. These pedagogical components were inconsistently reported across included studies, representing an important factor that may impact learning outcomes and should be addressed in future research [54].

### Limitations

This scoping review is limited by the heterogeneity of study designs, outcome measures, and learner populations. Most studies assessed simulation-based outcomes rather than clinical performance or patient-level endpoints. Additionally, the predominance of studies from high-income countries limits generalizability. Artificial intelligence applications remain underrepresented, and long-term skill retention and cost-effectiveness were infrequently evaluated.

## Conclusion

This scoping review demonstrates that extended reality technologies—particularly virtual reality—represent the most mature and widely adopted immersive tools in orthopedic surgical education. Across diverse training contexts, VR-based interventions were consistently associated with improvements in simulation-based technical performance, especially among medical students and early-stage residents [8, 17–20, 23]. Augmented reality applications were used more selectively, primarily in procedure-specific contexts such as preoperative planning and intraoperative guidance, supporting anatomical orientation and workflow preparation rather than comprehensive skills training [33–36].

In contrast, artificial intelligence applications remain emergent within orthopedic education and are currently limited to supportive roles, including information management, predictive analytics, and procedural planning assistance [16, 37, 41]. Robust empirical evidence evaluating AI-driven educational outcomes is scarce, highlighting the need for methodologically rigorous studies before broader curricular integration.

Overall, immersive technologies appear best positioned as adjuncts to traditional orthopedic training, offering standardized, reproducible environments that facilitate early skill acquisition and competency-based progression [4, 9, 26]. Future research should prioritize standardized outcome measures, long-term skill retention, cost-effectiveness analyses, and multicenter studies assessing transfer to clinical performance. Addressing these gaps will be essential to inform evidence-based integration of extended reality and artificial intelligence into orthopedic surgical curricula.

### Use of artificial intelligence tools

Artificial intelligence-based tools, including DeepL, Grammarly, Rayyan, and Springer Nature Author Services, were used to support translation, language editing, and article organization. These tools did not influence the interpretation of data or the scientific conclusions of the study.

### Ethical considerations

Since this review is based on previously published studies, ethical approval is not required. However, proper citation and acknowledgment of all original sources will be ensured, respecting copyright and intellectual property rights.

## Data Availability

No datasets were generated or analysed during the current study.
